# Geographical Detector-Based Spatial Modeling of the COVID-19 Mortality Rate in the Continental United States

**DOI:** 10.3390/ijerph18136832

**Published:** 2021-06-25

**Authors:** Han Yue, Tao Hu

**Affiliations:** 1 Center of GeoInformatics for Public Security, School of Geography and Remote Sensing, Guangzhou University, Guangzhou 510006, China; hanyue.geo@gmail.com; 2 Department of Geography, Oklahoma State University, Stillwater, OK 74078, USA; 3 Center for Geographic Analysis, Harvard University, Cambridge, MA 02138, USA

**Keywords:** COVID-19, geographical detector, spatial distribution, impact factor, interactive effect

## Abstract

Investigating the spatial distribution patterns of disease and suspected determinants could help one to understand health risks. This study investigated the potential risk factors associated with COVID-19 mortality in the continental United States. We collected death cases of COVID-19 from 3108 counties from 23 January 2020 to 31 May 2020. Twelve variables, including demographic (the population density, percentage of 65 years and over, percentage of non-Hispanic White, percentage of Hispanic, percentage of non-Hispanic Black, and percentage of Asian individuals), air toxins (PM2.5), climate (precipitation, humidity, temperature), behavior and comorbidity (smoking rate, cardiovascular death rate) were gathered and considered as potential risk factors. Based on four geographical detectors (risk detector, factor detector, ecological detector, and interaction detector) provided by the novel Geographical Detector technique, we assessed the spatial risk patterns of COVID-19 mortality and identified the effects of these factors. This study found that population density and percentage of non-Hispanic Black individuals were the two most important factors responsible for the COVID-19 mortality rate. Additionally, the interactive effects between any pairs of factors were even more significant than their individual effects. Most existing research examined the roles of risk factors independently, as traditional models are usually unable to account for the interaction effects between different factors. Based on the Geographical Detector technique, this study’s findings showed that causes of COVID-19 mortality were complex. The joint influence of two factors was more substantial than the effects of two separate factors. As the COVID-19 epidemic status is still severe, the results of this study are supposed to be beneficial for providing instructions and recommendations for the government on epidemic risk responses to COVID-19.

## 1. Introduction

The 2019 novel coronavirus disease (COVID-19) caused by the SARS-CoV-2 virus was declared as a pandemic on 11 March 2020, by the World Health Organization (WHO) [1]. Compared with Severe Acute Respiratory Syndrome (SARS), the COVID-19 disease is more infectious, so it has a broader range of outbreaks [2]. The disease has rapidly spread across the world and led to the infection or death of thousands of people. At present, the amounts of infections and deaths are still rising in many countries. Apart from the severe threat to human health, COVID-19 has also hammered economic development, social operation, and international relations [3].

The infectious COVID-19 disease has attracted extensive attention worldwide. Numerous studies have been carried out to explore the geographical distribution and the impact factors of the morbidity and mortality of the COVID-19 disease. Understanding how the transmission of COVID-19 is related to impact factors is critical to understanding the pandemic’s spatial pattern and intensity. Research shows that the transmission of the COVID-19 virus is dependent on numerous factors. Population density has been proved to be an important factor affecting the transmission of the virus. Therefore, compared with rural areas, urban areas usually suffer from more challenging situations; this is because urban areas are generally more densely populated. This phenomenon is not only found in the spread of the COVID-19 disease [4] but is also common among other types of diseases, such as the hand, foot, and mouth disease [5]. People’s physical conditions and lifestyle factors are also important factors affecting the severity of symptoms after infection. For instance, studies proved that smoking and obesity had associations for increased risks of COVID-19-related severity or fatality [2,6]. Meteorological conditions (e.g., average temperature, rainfall depth, relative humidity) during the same period of disease outbreak were found to influence the spread of viruses significantly. Specifically, Gupta et al. demonstrated that temperature and rainfall had a significant positive and negative association with the number of COVID-19 infections, respectively [4]. In a study conducted in China, Wang et al. pointed out that compared with other respiratory viruses, the spread of the COVID-19 virus was more likely to be affected by natural environment context features, such as temperature and humidity [2]. A similar study demonstrated that dry and cold weather was beneficial for the survival and transmission of droplet-mediated viral diseases; on the other hand, moist and warm weather conditions could weaken viral transmission [7]. The variation of weather conditions could also affect the COVID-19 disease; for example, the range of diurnal temperature change was positively associated with COVID-19 daily death counts [8]. Meteorological variables were also proved to affect the spread of other types of disease [9]. The climate variables’ influences on virus transmission may be due to their impacts on the vector population. Air quality is also an essential factor affecting the severity of COVID-19 cases. Wu et al. demonstrated that long-term air pollution exposure had a high probability of aggravating the health outcomes of COVID-19 cases [10].

Apart from the independent effects of various risk factors on the COVID-19 disease, complex interactive effects might exist between different risk factors. Previous research analyzed the independent influence of a single or a set of contextual factors on the COVID-19 incidence or mortality rate; however, the study on the interactive effects of two or more risk factors is very lacking. For example, physical environment features (e.g., meteorological conditions, air quality), people’s behavior and health conditions (e.g., age, sex, habits, immune health, etc.), and biological properties of viral infectivity have separate influences on human disease. More notably, their mutual interactions are also critical underlying factors. Specifically, Wu et al. demonstrated that people with certain underlying medical diseases and high air pollution exposure might suffer from higher mortality risks [10].

A variety of methods can assess the influences of risk factors, and the most common method is the multivariate regression model. However, these models have limitations, as they are usually premised on some assumptions, such as homoscedasticity and normality, and the violation of these assumptions will limit the model reliability. Additionally, if a study was not designed especially for evaluating the interaction effects, then incorporating interactions in the study will make it hard to assess the other effects [5], so it is necessary to develop an appropriate model for evaluating COVID-19 mortality.

The Geographical Detector technique is a novel method proposed by Wang et al. [11]. The principle of the Geographical Detector technique is based on the stratified spatial heterogeneity, i.e., the within-strata variance is less than the between-strata variance. This phenomenon is a fundamental characteristic of geographic events, and the different mechanisms in different stratum usually cause it. The Geographical Detector is a new tool to measure, mine, and utilize the stratified spatial heterogeneity. Its theoretical core is to detect the consistency of spatial distribution patterns between dependent variables and independent variables, through which the explanatory degrees of independent variables to dependent variables can be determined. Suppose a geographical event is affected by a particular factor; in that case, this factor’s spatial distribution pattern will then be comparable to that of the geographical event [11]. The consistent spatial distribution patterns of the geographical event and the factor indicate that the factor could promote or inhibit the occurrence of the geographical event. Similarly, in our study, if a risk factor could significantly affect the COVID-19 fatality rate, their spatial stratifications may be more consistent.

The Geographical Detector technique is novel. It extracts the implicit interrelationships between risk factors and health events without any assumptions or restrictions regarding explanatory and response variables, challenging the model that uses classic epidemiological methods. Additionally, the method applies to both quantitative and nominal data; the latter may cause trouble with traditional regression when there are too many categories. In summary, the Geographical Detector provides a quick, easy, and efficient way for researchers to grasp associations between human health and risk factors from the perspective of spatial distribution [12].

This study assessed the associations between COVID-19 mortality and a series of risk factors using the recently proposed Geographical Detector technique [11]. We first mapped and investigated the spatial distribution patterns of the COVID-19 mortality rate at the county level. Then, we reported on other impact factors, including socio-demographic, economic, air quality, climate, and behavior and comorbidity factors. Lastly, we applied the four detectors to evaluate the relationships between COVID-19 mortality and those factors and analyzed and discussed the results.

## 2. Materials and Methods

### 2.1. Spatial Empirical Bayes-Smoothed COVID-19 Fatality Rate

The COVID-19 mortality data used in this study were sourced from Johns Hopkins University, Center for Systems Science and Engineering Coronavirus Resource Center [13]. A death on February 6 is thought to be the earliest death connected to SARS-CoV-2 (the virus which causes COVID-19) in the US [14]. To obtain a reliable understanding of the risk of dying from COVID-19, we collected the count of persons who died from COVID-19 in each county between 6 February 2020 and 31 May 2020 in the continental United States. The crude mortality rate is then the ratio of the number of dead COVID-19 cases to the total population.

In the field of public health, the crude fatality rate is usually estimated as the death cases/population ratio, and the estimation accuracy mainly depends on the count of the denominator. Compared with geographical units with large populations, geographical units with small populations are likely to produce less accurate rate calculations. Differences in population size may cause the problem of variance instability and spurious outliers in the raw rates, making the raw rates inadequate to represent the relative magnitude of the underlying risks. Spatial empirical Bayes (SEB) smoothing is a common technique to avoid using these crude rates to evaluate the actual rates [15]. The basic principle of SEB smoothing is to improve the crude rate’s precision by borrowing strength from neighboring observations [16]. It is implemented by calculating a weighted average between each geographical unit’s crude rate and the regional average, with weights proportional to the underlying populations at risk. The rates of geographical units with small populations are inclined to be significantly adjusted, while the rates of geographical units with larger populations will barely change [17,18].

Let *r_i_* = *y_i_*/*n_i_* be the observed rate of geographical unit *i*, where *y_i_* represents the count observed, and *n_i_* means population size. The unknown rate in the geographical unit *i* is denoted as $\lambda_{i}$. Assume the underlying rates are independent samples from a prior probability distribution, with mean $\mu_{i}$ and variance $\varphi_{i}$. The SEB smoothing ${\hat{\lambda }}_{i}$ of $\lambda_{i}$ is a weighted combination of *r_i_* and $\mu_{i}$ [19]:

(1) $${\hat{\lambda }}_{i}=w_{i}r_{i}+\left(1-w_{i}\right)\mu_{i}$$

depends on $\varphi_{i}$, $\mu_{i}$, and *n_i_*:

(2) $$w_{i}=\frac{\varphi_{i}}{\varphi_{i}+\frac{\mu_{i}}{n_{i}}}\;$$

The prior distribution is generally assumed to follow the Gamma distribution (mean μ=ν/α, variance $\varphi =v/\alpha^{2}$). Therefore, μ and φ can be empirically estimated from the data observed on the set of geographical units in the neighboring regions:

(3) $$\hat{\mu }=\frac{\sum y_{i}}{\sum n_{i}}$$

(4) $$\hat{\;\varphi }=\frac{\sum n_{i}{\left(r_{i}-\hat{\mu }\right)}^{2}}{\sum n_{i}}-\frac{\hat{\mu }}{\bar{n}}\;$$

The SEB smoothing estimator ${\hat{\lambda }}_{i}$ of rate $\lambda_{i}$ is then:

(5) $${\hat{\lambda }}_{i}=\hat{\mu }+\frac{\hat{\varphi }\left(r_{i}-\hat{\mu }\right)}{\hat{\varphi }+\frac{\hat{\mu }}{n_{i}}}\;$$

As the raw mortality rates of COVID-19 are tiny numbers, we transformed them into rates per 100,000 people. Then, the adjusted mortality rates were generated using the SEB smoothing method.

### 2.2. Data of Explanatory Variables

COVID-19 mortality is determined by diverse and complex factors. Some factors are related to the transmission of the virus. Demographic characteristics are a category of important factors that could affect the transmission of COVID-19 [20]. Therefore, we considered the influence of population density, which reflects the virus transmission environment. Additionally, the racial/ethnic disparity of COVID-19 attack rates was found by many researchers [20,21]. This study considered the population’s racial/ethnic compositions (the proportion of non-Hispanic White, proportion of non-Hispanic Black, proportion of Hispanic, and proportion of Asian individuals). Ambient climate conditions could affect the transmission and survival of coronaviruses [22], so we obtained the average accumulated precipitation, the average relative humidity, and the average temperature across 1 January 2020–31 May 2020. Some factors are related to the mortality of the disease. Previous studies revealed that the risk of COVID-19 mortality is highest for older people [23], so the proportion of people over 65 was considered as another impact factor in this study. An increase in the exposure to particulate matter (PM) 2.5, a typical air toxin, was proven to increase the COVID-19 death rate [10]. In this study, we collected the PM2.5 levels across the period 2000–2018 and obtained the average as the representation of the long-term exposure to PM2.5. Some human behaviors (e.g., smoking) and comorbidities (e.g., cardiovascular) could increase the likelihood of severe outcomes during infectious disease outbreaks, so we considered the percentage of adults that reported smoking in 2019 and the cardiovascular death rate with an age of 65 and over (2016–2018).

In summary, we collected a variety of 12 demographic, air toxins, climate, and behavior and comorbidity factors which may affect the COVID-19 fatality rate. Table 1 lists the names, descriptions, and data sources of these variables.

### 2.3. Method: Geographical Detector

The Geographical Detector technique is designed to evaluate the associations between a geographical phenomenon and relevant risk factors. It is based on the spatial variance analysis, the underlying principle of which is to estimate the consistencies between the spatial distribution patterns of the studied geographical event (e.g., COVID-19 mortality rate) and those of potential risk factors (e.g., population density). We assume that a geographical event would show a similar spatial distribution pattern with that of a risk factor if the risk factor’s attribute significantly impacts the geographical event’s occurrence [11].

Specifically, the Geographical Detector method first needs to divide the spatial distributions of the geographic event and risk factors into subregions according to their spatial stratified heterogeneity. Spatial heterogeneity is a major feature of the geographic phenomenon, and it refers to the uneven distributions of events across a region, or, simply, the spatial variation of attributes [24,25]. A stratification of heterogeneity is, essentially, a segmentation of a research region, where observations are homogeneous within each stratum but not between strata [26]. The spatial heterogeneity between areas (each area consists of one or more units) is commonly referred to as spatial stratified heterogeneity, a common phenomenon, such as climate or ecological zones, spatial variability of soil types, and land-use patterns [26]. If the attributes within the strata are uniform or the variances within the strata are zeros, the stratified heterogeneity is mostly significant; on the contrary, the stratification of heterogeneity will disappear if there is no difference between the strata. Generally, a stratification of heterogeneity partitions a target population by minimizing the within-strata variance and maximizing the between-strata variance of an attribute. Technically, the stratification of heterogeneity can be accomplished by either prior knowledge or classification methods [11]. Therefore, the consistency between the spatial stratified heterogeneities of a pair of geographic events suggests the possibility that there is a statistical association between these phenomena [27].

The goal of the Geographical Detector model is to assess whether spatial stratified heterogeneity exists for a geographic phenomenon and explore the interpretation of a geographic phenomenon by comparing the spatial coincidence of its strata against the strata of suspected determinants. Specifically, the Geographical Detector model puts forward four detectors (i.e., a factor detector, risk detector, ecological detector, and interactive detector) to determine the main and interactive effects of possible factors on the examined geographic event. A factor detector is used to determine which factors are responsible for the incidence of the studied geographic event. A risk detector can be applied to recognize the geographical area with a high possibility of the occurrence of the event. An ecological detector is used to assess whether the influences of various factors on the studied geographic event differ remarkably from each other. An interactive detector is applied to determine whether multiple factors independently or dependently affect the occurrence of the studied event [5].

Spatial stratified heterogeneity is likely to indicate that there may be distinct mechanisms in strata [28], which may be obscured or even cause aggregation bias and ecological fallacy by global models [29,30]. The Geographical Detector technique is novel as it extracts the underlying mutual associations between a studied geographic event and suspected factors, without any restrictions on the response and explanatory variables [5,31]. Additionally, the Geographical Detector method is suitable for quantitative and qualitative data, while traditional regression models may incur problems when the nominal data has too many categories [32].

**Figure 1:**
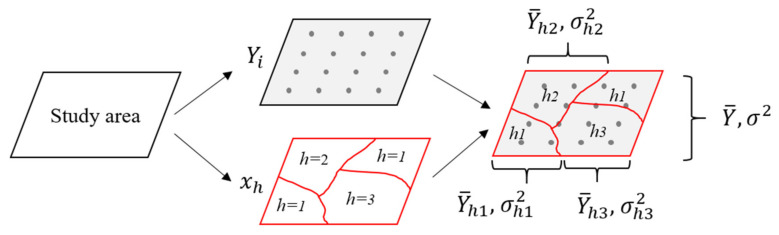
The principle of the Geographical Detector.

This study assumes that the study area is stratified by a potential risk factor *X* into subregions (*x_1_*, *x_2_*, *x_3_*) in the geographical space (Figure 1). The risk factor layer overlays the spatial distribution of the COVID-19 fatality rate. The averages and variances of fatality rates in each subregion and the whole study area are, respectively, represented as ${\bar{Y}}_{h1},{\bar{Y}}_{h2},{\bar{Y}}_{h3},\bar{Y}$ and $\sigma_{h1}{}^{2},\;\sigma_{h2}{}^{2},\;\sigma_{h3}{}^{2},\;\sigma^{2}$. If the COVID-19 mortality rate is wholly determined by factor *D*, the rates will be identical everywhere in each of the subregions (*x_1_*, *x_2_*, *x_3_*), and $\sigma_{h1}{}^{2},\;\sigma_{h2}{}^{2},\;\mathrm{and}\;\sigma_{h3}{}^{2}$ will be zeros. On the contrary, if the COVID-19 mortality rate is entirely independent of *X*, the accumulated variance within the subregions will be consistent with the whole study area’s pooled variance. This mechanism is measured by the Power of Determinant (*PD*) [26]:

(6) $$PD=1-\frac{\sum_{h=1}^{L}\sum_{i=1}^{N_{h}}{\left(Y_{hi}-{\bar{Y}}_{h}\right)}^{2}}{\sum_{i=1}^{N}{\left(Y_{i}-\bar{Y}\right)}^{2}}=1-\frac{\sum_{h=1}^{L}N_{h}\sigma_{h}{}^{2}}{N\sigma^{2}}$$

where a study area consists of *N* units and is stratified into *L* stratums by a factor, represented as *h* = 1, 2, …, *L*, respectively; stratum *h* is comprised of *N_h_* units; *Y_i_* and *Y_hi_*, respectively, denote the value of unit *i* and stratum *h*; ${\bar{Y}}_{h}=\left(1/N_{h}\right)\overset{N_{h}}{\underset{i=1}{\sum }}Y_{hi}$ represents the stratum mean; $\sigma_{h}{}^{2}=\left(1/N_{h}\right)\overset{N_{h}}{\underset{i}{\sum }}{\left(Y_{hi}-{\bar{Y}}_{h}\right)}^{2}$ represents the stratum variance; $\bar{Y}=\left(1/N\right)\overset{N}{\underset{i=1}{\sum }}Y_{i}$ represents the population mean; $\sigma^{2}=\left(1/N\right)\overset{N}{\underset{i}{\sum }}{\left(Y_{i}-\bar{Y}\right)}^{2}$ represents the population variance. The ***PD*** quantifies the degree to which a risk factor explains the COVID-19 mortality, and its value lies between 0 and 1. The more considerable the amount of *PD*, the greater the influence of the factor. If a factor completely controls the COVID-19 mortality rate, *PD* = 1; if it is entirely unrelated to the COVID-19 mortality rate, *PD* = 0.

The Geographical Detector method is based on the *PD*, which generates the following four detectors [11].

#### 2.3.1. Risk Detector

The risk detector aims to determine whether the mean fatality rates in each subregion are statistically different from each other when a potential risk factor *X* stratifies the study area. This is achieved by the *t*-value test [31]:

(7) $$t_{{\bar{Y}}_{h1}-{\bar{Y}}_{h2}}=\frac{{\bar{Y}}_{h1}-{\bar{Y}}_{h2}}{\sqrt{\frac{\sigma_{h1}{}^{2}}{N_{h1}}+\frac{\sigma_{h2}{}^{2}}{N_{h2}}}}\;$$

where ${\bar{Y}}_{hi}$, $\sigma_{hi}{}^{2}$, and *N_hi_* denotes the mean fatality rate, the variance of fatality rate, and sample size in subregion *h_i_*, respectively.

The *t*-value follows approximately a Student’s *t* distribution, with the degree of freedom (*df*) as:

(8) $$df=\frac{\frac{\sigma_{h1}{}^{2}}{N_{h1}}+\frac{\sigma_{h2}{}^{2}}{N_{h2}}}{\frac{1}{N_{h1}-1}{\left[\frac{\sigma_{h1}{}^{2}}{N_{h1}}\right]}^{2}+\frac{1}{N_{h2}-1}{\left[\frac{\sigma_{h2}{}^{2}}{N_{h2}}\right]}^{2}}\;$$

If the null hypothesis $H_{0}:{\bar{Y}}_{h1}={\bar{Y}}_{h2}$ is rejected at the confidence level α (usually 5%), there is a significant difference between mortality rates of two subregions.

#### 2.3.2. Factor Detector

The factor detector quantifies to which extent a factor explains the dependent variable’s spatial variance, which is evaluated by the *PD*, as shown in Formula (6).

#### 2.3.3. Ecological Detector

The ecological detector is used to explore whether the impacts of two factors *X_1_*, *X_2_*, on the dependent variable have a significant difference, and it is determined by the *F* statistics [31]:

(9) $$F=\frac{N_{X1}\left(N_{X1}-1\right)SSW_{X1}}{N_{X2}\left(N_{X2}-1\right)SSW_{X2}}\;$$

(10) $$SSW_{X1}=\overset{L1}{\underset{h=1}{\sum }}N_{h}\sigma_{h}{}^{2},SSW_{X2}=\overset{L2}{\underset{h=1}{\sum }}N_{h}\sigma_{h}{}^{2}\;$$

where *N_X1_* and *N_X2_*, respectively, denote the sample size within the coverage of *X_1_* and *X_2_*. *SSW_X1_* and *SSW_X2_* represent the sum of the within strata variances when *X_1_* and *X_2_*, respectively, form the strata. *L_1_* and *L_2_*, respectively, represent the strata number of *X_1_* and *X_2_*.

If the null hypothesis *H_0_*: *SSW_X1_* = *SSW_X2_* is rejected at the confidence level α (usually 5%), the influences of *X_1_* and *X_2_* on the dependent variable are considered to be statistically significant.

#### 2.3.4. Interactive Detector

The interactive detector is used to quantify the interaction effect between the impacts of two factors, e.g., *X_1_* and *X_2_*, on the dependent variable [31]. This is achieved by firstly overlapping the geographical layers of *X_1_* and *X_2_* to form a new layer *X_3_* and obtaining the attributes of layer *X_3_* by combining layer *X_1_* and *X_2_*. Then, by comparing the *PD* of layer *X_3_* with those of *X_1_* and *X_2_*, the interactive detector could determine whether two factors, *X_1_* and *X_2_*, when taken together, have stronger or weaker influences on the COVID-19 mortality rate than they do independently.

We can classify the interaction effects between two factors as:

Enhance: if *PD*(*X_1_*∩*X_2_*) > *PD*(*X_1_*) or *PD*(*X_2_*);
Enhance, bivariate: if *PD*(*X_1_*∩*X_2_*) > *PD*(*X_1_*) and *PD*(*X_2_*);
Enhance, nonlinear: if *PD*(*X_1_*∩*X_2_*) > *PD*(*X_1_*) + *PD*(*X_2_*);
Weaken: if *PD*(*X_1_*∩*X_2_*) < *PD*(*X_1_*) + *PD*(*X_2_*);
Weaken, univariate: if *PD*(*X_1_*∩*X_2_*) < *PD*(*X_1_*) or *PD*(*X_2_*);
Weaken, nonlinear: if *PD*(*X_1_*∩*X_2_*) < *PD*(*X_1_*) and *PD*(*X_2_*);
Independent: if *PD*(*X_1_*∩*X_2_*) = *PD*(*X_1_*) + *PD*(*X_2_*).

## 3. Results

**Figure 2:**
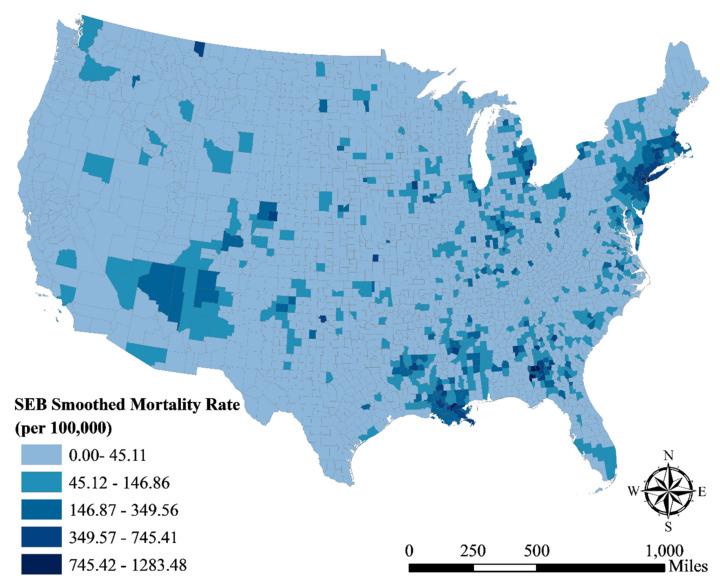
A map with COVID-19 mortality rates smoothed by the spatial empirical Bayes method.

The SEB-smoothed COVID-19 mortality rate in the US is shown in Figure 2. As we can see from the map, the spatial heterogeneity of the mortality rate is large. The rate ranges from 0 to 1283.48, with a mean of 40.73 (per 100,000 people). Among 3108 counties, the fatality rates in 998 counties are less than 10 per 10,000 people, accounting for 32%. The global Moran’s *I* of the COVID-19 fatality rate is 0.62 (*p* < 0.001), which indicates a strong positive spatial autocorrelation. Counties with high fatality rates are mainly located in the eastern area, such as New York, Philadelphia, and Washington DC in the north-east, Louisiana, Alabama, and Georgia in the south, and Indiana and Michigan in the north. Besides, some counties in Colorado, Arizona, California, and Washington also have high fatality rates.

**Figure 3:**
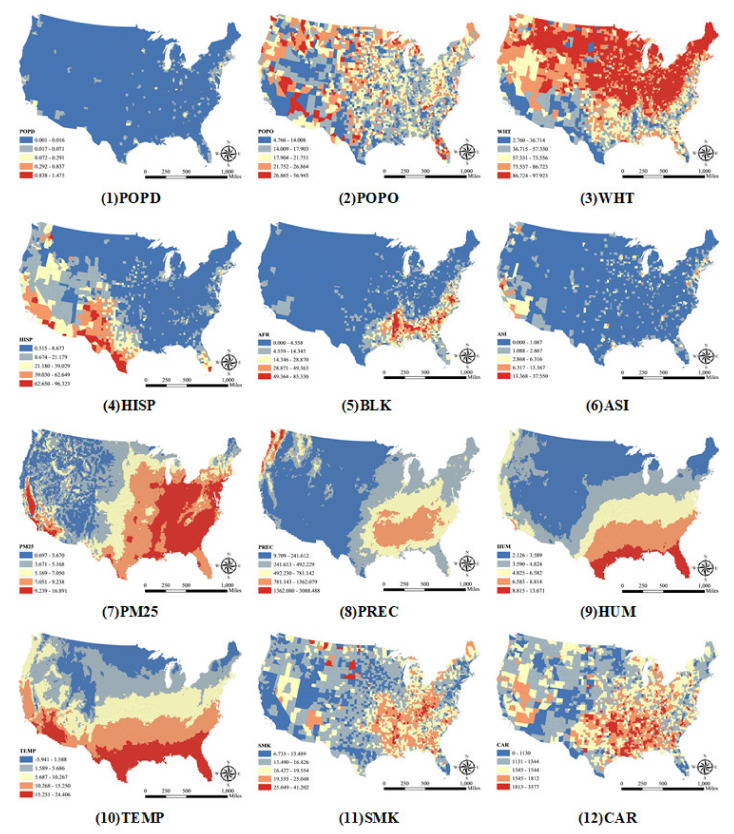
Maps of explanatory variables. POPD: population density; POPO: percentage of 65 years old and over; WHT: percentage of non-Hispanic White individuals; HISP: percentage of Hispanic individuals; BLK: percentage of non-Hispanic Black individuals; ASI: percentage of Asian individuals; PM25: average level of PM2.5; PREC: average accumulated precipitation; HUM: average relative humidity; TEMP: average temperature; SMK: percentage of adults that reported currently smoking in 2019; CAR: cardiovascular death rate.

Continuous variables should be discretized to obtain the layers’ corresponding stratifications to apply the Geographical Detector method. This was achieved by using the Jenks Natural Breaks classification method, which groups similar values and, at the same time, maximizes the differences between groups. Figure 3 shows the classifications of the explanatory variables used in this study. As we can see, the distributions of demographic, air toxins, climate, behavior and comorbidity factors vary significantly across space. For instance, the maximum and minimum values of non-Hispanic White (WHT) percentages are very different: in some counties, the value is 2.76%, while in other counties, it reaches 97.923%. As for PM2.5, the values range from 0.697 to 16.891 μg/m^3^. The differences in climate factors and behavior and comorbidity conditions between counties are also large. For example, some counties receive an average precipitation of 9.709 mm, while some counties receive up to 3088.488 mm. The average percentage of adult smokers is 17.837%, and the minimum and maximum are 6.735% and 41.202%.

The results of the Geographical Detector are listed in Table 2 (risk detector), Table 3 (factor detector), Table 4 (ecological detector), and Table 5 (interactive detector). The risk detector analyzes the influence of various factors on the COVID-19 mortality rate.

Table 2 presents each subregion’s average mortality rate when a corresponding explanatory variable stratifies the study area. As we can see, when the population density (POPD) is high, the COVID-19 mortality rate is also high, especially when the population density is higher than 0.837 (per 100,000 people/km^2^), the mean COVID-19 mortality rate is 1016.509 (per 100,000 people). This finding means that there is a correlation between population density and the COVID-19 mortality rate. The COVID-19 mortality rate also becomes larger with the increase in BLK and ASI. As to WHT, the COVID-19 mortality rate decreases as a whole while the factor increases. When WHT is less than 36.715%, the average COVID-19 mortality rate is only 42.877 per 100,000 people. Overall, the average COVID-19 mortality rate rises gradually with the increase in the PM2.5 level. The correlations between the average COVID-19 mortality rate and the rest of the factors can be analyzed in the same way based on the results of the risk detector.

Based on the *PD* value, the factor detector reveals the extent to which a factor explains the variation of the COVID-19 fatality rate. Table 3 shows that population density (POPD) explains the spatial variability of the COVID-19 mortality rate to the maximum extent, followed by BLK, WHT, PM25, POPO, TEMP, ASI, HUM, SMK, PREC, and HISP, while CAR has the least influence.

The ecological detector identifies the difference between the values of two *PD*s; in other words, the difference between the influences of two factors on the explained variable. Table 4 shows that the differences between the *PD* of population density (POPD) and *PD*s of the rest of the factors are all statistically significant. As to the *PD* of the old population (POPO), it is significantly different from those of HISP, BLK, PREC, SMK, and CAR, but it is not substantially different from WHT, ASI, PM25, HUM, and TEMP. The differences between *PD*s of the rest factors could be interpreted in the same way.

The interactive detector determines the interaction effects between pairs of *PD*s. The results shown in Table 5 are impressive, as the interaction effects are either “enhance, bivariate” or “enhance, nonlinear”. This means that the joint impacts of two factors on the COVID-19 mortality rate measured by the *PD* are more substantial than the effects of two separate factors. For example, the interactive *PD* of POPD and POPO is 0.142, which is higher than *PD*s of two sole factors, POPD (0.094) and POPO (0.033). Additionally, the interactive effect is stronger than the sum of two individual effects, so the interactive effect between POPD and POPO is “enhance, nonlinear”. The interactive *PD* of POPO and WHT is 0.068, which is higher than *PD*s of two sole factors, POPO (0.033) and WHT (0.044). However, the interactive effect is weaker than the sum of two individual effects, so the interactive effect between POPO and WHT is “enhance, bivariate”.

## 4. Discussion

The Geographical Detector method is novel as it extracts the associations between the observed process and possible influencing factors by the consistency of their spatial distribution patterns. It is an efficient tool and easy to implement. This study applied four geographical detectors to analyze the effects of demographic, air toxins, climate, behavior, and comorbidity factors on the COVID-19 mortality rate. The aim was to determine the differences of the degrees at which different factors influence the spatial distribution of the COVID-19 mortality rate and the interaction effects between different factors.

Firstly, we focused on which factors play more critical roles in the COVID-19 mortality rate. According to the results of four geographical detectors, population density (POPD) and percentage of non-Hispanic Black individuals (BLK) were the two most important factors responsible for the COVID-19 mortality rate. The higher the POPD (*PD* = 0.094) is, the higher the mortality rate is; the same is true for BLK (*PD* = 0.074). In general, places with higher population densities have more frequent interpersonal contact, enhancing virus transmission in the local area and increasing infection chances [33]. Therefore, POPD was positively associated with the COVID-19 mortality rate in American counties. Previous research demonstrated that counties with higher percentages of black residents had more COVID-19 diagnoses and deaths; this result is consistent with ours. The reason may be that people in these places have a higher prevalence of comorbidities and are exposed to more massive air pollution, which increases the risk of becoming seriously ill or even death when they are infected with the COVID-19 virus [34]. Compared with POPD and BLK, the percentage of non-Hispanic White individuals (WHT), the average level of particulate matter (PM) 2.5 (PM25), percentage of individuals 65 years and over (POPO), average temperature (TEMP), and percentage of Asian individuals (ASI) had relatively small impacts on the COVID-19 mortality rate. As to PM2.5 (*PD* = 0.043), Zhu et al. found a significant positive relationship between the exposure level to air pollutants (such as PM2.5, PM10, NO2, and O3) and the number of COVID-19 confirmed cases [35]. More specifically, PM2.5 is a significant factor influencing both the natural environment and population health [36]. The COVID-19 death rate will increase by 8% when the PM2.5 level increases only by 1 μg/m^3^ [10]. The elderly usually suffer from certain underlying medical conditions, especially chronic illnesses such as diabetes, hypertension, heart and respiratory disease, etc. These comorbidities will increase the death risk when a person becomes infected with the COVID-19 virus [37]. This may explain the correlation between POPO (*PD* = 0.033) and the COVID-19 mortality rate. The factor detector showed that the *PD* value of TEMP was 0.025. The near-surface air temperature has been demonstrated to be an essential factor affecting the survival and spread of droplet-mediated viral diseases, such as influenza [7]. This is because the temperature in the same period could affect the vectors’ population; therefore, it influences the transmission of the virus [38]. Previous studies have shown a positive linear relationship between the mean temperature and the amount of COVID-19 cases with a threshold of 3 °C [31]. The average relative humidity (HUM), percentage of adults that reported smoking (SMK), average accumulated precipitation (PREC), percentage of Hispanic individuals (HISP), and cardiovascular death rate (CAR) had less impact on the COVID-19 mortality rate compared with other factors, while CAR had the most minimal effect. The *PD* value of HUM in this study was 0.019. A humid environment is not conducive to viral transmission [7]; this is a likely explanation why Ma et al. found that the relative humidity and COVID-19 daily death count were negatively associated with each other [8]. For the influence of smoking, Brake revealed that smoking could impair our ability to combat COVID-19 [39]; similarly, Taghizadeh-Hesary demonstrated that smoking had an adverse effect on the health outcomes of COVID-19 patients because of the possible decreased immune response [40]. However, for our study, the influence of smoking (*PD* = 0.015) on the mortality rate of COVID-19 was not as significant as the factors mentioned above, which is in line with Mollalo [20]. One reason may be that, in addition to traditional cigarettes, there are many other under-investigated smoking methods and devices, such as electronic cigarettes and waterpipe smoking [39]. The factor detector showed that the *PD* value of PREC was 0.013. Precipitation has been demonstrated to be a deterrent to the transmission of COVID-19 transmission [2]; the negative influence of rainfall on the number of COVID-19 infections has also been observed [4]. Zheng et al. found that people who had comorbidities such as cardiovascular disease were more likely to experience cardiac insufficiency and sudden deterioration in their conditions after they got infected with the COVID-19 virus. Therefore, the proportion of patients with underlying cardiovascular diseases (*PD* = 0.004) may increase the COVID-19 death rate [41]. The influences of the population’s racial compositions on the COVID-19 mortality rate were notably different. The factor detector showed that the *PD* values of BLK, WHT, ASI, and HISP were 0.074, 0.044, 0.025, and 0.012, respectively. Therefore, compared to the proportion of non-Hispanic Black individuals, the proportion of non-Hispanic White, proportion of Hispanic, and proportion of Asian individuals had weaker influences on the COVID-19 mortality rate.

Through the interactive detector, we examined the interaction effects between pairs of factors. The results demonstrated that these interactive effects were either “enhance, bivariate” or “enhance, nonlinear”. Therefore, for any two factors considered in this study, they had a more substantial influence on the COVID-19 mortality rate when they were taken together than they did independently. For example, the interaction of SMK (*PD* = 0.015) and PM25 (*PD* = 0.043) nonlinearly enhanced the COVID-19 mortality rate, which was 0.064. The reason for this finding may be that when a proper smoking rate is combined with the average level of PM2.5, patients’ risks of suffering severe or deteriorating conditions after they become infected with the COVID-19 virus are more likely to increase. The influence of CAR was small, but the interactive effects of CAR and other factors were large. For instance, the interaction of CAR (*PD* = 0.004) and PM25 (*PD* = 0.043) nonlinearly enhanced the COVID-19 mortality rate, which was 0.051. The underlying cause may be that both CAR and PM25 could increase the likelihood of severe outcomes; when these two factors were combined, the adverse effect was enhanced. The interaction of POPO and POPD also nonlinearly increased the COVID-19 mortality rate, which was 0.142. Therefore, when a proper proportion of older people was combined with population density, the death rate of the COVID-19 disease was increased. Previous studies demonstrated that a larger population density meant a better environment for virus transmission, and the risk of death among the elderly with COVID-19 disease was the highest. Therefore, the combination of these two factors was likely to make the situation much worse. The interaction of TEMP (*PD* = 0.025) and HUM (*PD* = 0.019) bivariately enhanced the COVID-19 mortality rate, which was 0.043. The cause might be that when a proper temperature is combined with relative humidity, the virus is more survivable and capable of reproducing and transmitting. Similarly, the interaction of PM25 (*PD* = 0.043) and POPD (*PD* = 0.094) bivariately enhanced the COVID-19 mortality rate, which was 0.118. Therefore, population density and level of PM2.5 could reinforce each other’s influence on the COVID-19 mortality rate.

This study has implications for future research. Firstly, existing research mostly focused on the independent effects of various risk factors on the COVID-19 mortality; scanty attention had been paid to the interactive effect between different risk factors. As for two risk factors, we need to examine their individual influences and understand their interactive effect. Secondly, the results presented in this research could offer a reference for understanding the spatial distribution patterns and epidemiological characteristics of the COVID-19 mortality rate. Finally, implications from this study provide clues for policymakers to develop strategies to prevent and control the COVID-19 epidemic; for example, high priority should be paid to regions with high population densities and large proportions of older people.

One limitation of this study is the discretization of quantitative data. The Geographical Detector method requires a discretization of the impact factors before they are input into the model. For qualitative data, it is easy to obtain their classifications according to their categorical attributes. We used a clustering method for discretization for quantitative data as we had no prior knowledge about these variables. Clustering methods, however, tend to be arbitrary; therefore, variable discretization using these clustering methods may weaken the Geographic Detector’s ability to characterize the actual associations between COVID-19 mortality rate and risk factors. The problem of how to discretize quantitative data effectively should be considered in future studies.

## 5. Conclusions

This research examined the spatial distribution pattern of the COVID-19 mortality rate in the continental US. Results showed that the COVID-19 mortality rate is heterogeneous in the US, and it is highly autocorrelated in space; a large proportion of the counties with high mortality rates are distributed in the eastern area. Furthermore, using the novel Geographical Detector method, we investigated the potential determinants of the COVID-19 mortality rate. This study’s findings suggest that population density and percentage of non-Hispanic Black individuals played a much larger role in affecting the COVID-19 mortality rate compared with other studied factors. The joint impacts of pairs of factors are also presented and can be compared with their separate impacts. What is notable is the interactive effect between different factors: since all the interactive effects promoted the values of the Power of Determinant, combinations of the studied factors will be more effective at explaining the spatial variability of the COVID-19 mortality rate when compared with separate factors. Most existing research considered the independent effects of various factors on the COVID-19 disease; however, the causes of COVID-19 mortality are complex. This study demonstrated that the Geographical Detector technique could measure not only the separate effects of two or even more factors on the COVID-19 mortality rate but also the interactive effect between different factors.

## Figures and Tables

**Table 1:** Explanatory variables used in this study, together with descriptions and data sources.

Category	Name	Description	Source
Demographic	(1) POPD (2) POPO (3) WHT (4) HISP (5) BLK (6) ASI	(1) Population density (total population for each county/land area of the corresponding county) (2) Percentage of 65 years and over (3) Percentage of non-Hispanic White (4) Percentage of Hispanic (5) Percentage of non-Hispanic Black (6) Percentage of Asian ((2)–(6) assumed proportion to the fraction of the population living in the county)	US Census Bureau Population Estimates 2018 (https://www.census.gov/programs-surveys/acs/data.html, (accessed on 10 July 2020))
Air toxins	(7) PM25	(7) Average level of particulate matter (PM) 2.5 (0.01° × 0.01° grid resolution PM2.5 prediction, averaged across the period 2000‒2018)	Atmospheric Composition Analysis Group (https://sites.wustl.edu/acag/datasets/surface-pm2-5/, (accessed on 10 July 2020))
Climate	(8) PREC (9) HUM (10) TEMP	(8) Average accumulated precipitation (9) Average relative humidity (10) Average temperature ((8)–(10): 4 km × 4 km grid resolution climate predictions, averaged across the period 1 February 2020—31 May 2020)	Climate Engine (https://clim-engine.appspot.com/climateEngine, (accessed on 10 July 2020))
Behavior and comorbidity	(11) SMK (12) CAR	(11) Percentage of adults that reported currently smoking in 2019	County Health Rankings and Roadmaps (https://www.countyhealthrankings.org/, (accessed on 10 July 2020))
		(12) Cardiovascular death rate per 100,000 with an age of 65 and over (2016–2018)	CDC’s Interactive Atlas of Heart Disease and Stroke (https://nccd.cdc.gov/DHDSPAtlas/Default.aspx, (accessed on 10 July 2020))

**Table 2:** Results of the risk detector.

Variable	Stratum	Range of a Factor’s Values in Each Stratum				
	Mortality	Average Mortality Rate in Each Stratum				
POPD	Stratum	<0.017	0.017–0.071	0.072–0.291	0.292–0.837	>0.837
	Mortality	25.798	86.073	147.684	371.756	1016.509
POPO	Stratum	<14.009	14.009–17.903	17.904–21.751	21.752–26.864	>26.864
	Mortality	39.127	45.341	25.413	13.367	13.169
WHT	Stratum	<36.715	36.715–57.330	57.331–73.556	73.557–86.723	>86.723
	Mortality	42.877	58.424	41.439	24.374	16.300
HISP	Stratum	<8.674	8.674–21.179	21.180–39.029	39.030–62.649	>62.649
	Mortality	26.239	43.897	30.530	25.299	12.207
BLK	Stratum	<4.559	4.559–14.345	14.346–28.870	28.871–49.363	>49.363
	Mortality	20.881	46.247	54.274	85.210	120.908
ASI	Stratum	<1.088	1.088–2.867	2.868–6.316	6.317–13.367	>13.367
	Mortality	23.725	32.296	56.225	60.282	81.583
PM25	Stratum	<3.671	3.671–5.168	5.169–7.050	7.051–9.238	>9.238
	Mortality	26.181	16.275	20.338	33.146	58.011
PREC	Stratum	<241.613	241.613–492.229	492.230–781.142	781.143–1362.079	>1362.079
	Mortality	21.159	37.004	36.021	40.144	29.405
HUM	Stratum	<3.590	3.590–4.824	4.825–6.582	6.583–8.814	>8.814
	Mortality	22.622	35.659	22.756	44.674	56.282
TEMP	Stratum	<1.589	1.589–5.686	5.687–10.267	10.268–15.250	>15.250
	Mortality	14.389	24.143	40.740	27.995	48.511
SMK	Stratum	<13.490	13.490–16.426	16.427–19.554	19.555–25.048	>25.048
	Mortality	38.280	24.024	25.609	45.190	13.845
CAR	Stratum	<1131	1131–1344	1345–1544	1545–1812	>1812
	Mortality	38.068	26.928	26.655	31.880	37.797

Note: average of the explained variable (COVID-19 mortality rate) according to the stratums of each explanatory variable.

**Table 3:** Results of the factor detector.

Variable	*PD*	*p*-Value
POPD	0.094	<0.001
BLK	0.074	<0.001
WHT	0.044	<0.001
PM25	0.043	<0.001
POPO	0.033	<0.001
TEMP	0.025	<0.001
ASI	0.025	<0.001
HUM	0.019	<0.001
SMK	0.015	<0.001
PREC	0.013	<0.001
HISP	0.012	<0.001
CAR	0.004	<0.001

Note: sorted by *PD*.

**Table 4:** Results of the ecological detector.

	POPD	POPO	WHT	HISP	BLK	ASI	PM25	PREC	HUM	TEMP	SMK
POPO	Y										
WHT	Y	N									
HISP	Y	Y	Y								
BLK	Y	Y	Y	Y							
ASI	Y	N	Y	N	Y						
PM25	Y	N	N	Y	Y	Y					
PREC	Y	Y	Y	N	Y	N	Y				
HUM	Y	N	Y	N	Y	N	Y	N			
TEMP	Y	N	Y	N	Y	N	Y	N	N		
SMK	Y	Y	Y	N	Y	N	Y	N	N	N	
CAR	Y	Y	Y	N	Y	Y	Y	N	N	Y	N

Note: Y means the difference between the influences of two factors on the COVID-19 mortality rate is statistically significant with 95% confidence, and N means not.

**Table 5:** Results of the interactive detector.

	POPD	POPO	WHT	HISP	BLK	ASI	PM25	PREC	HUM	TEMP	SMK
POPO	0.142										
WHT	0.144	*0.068*									
HISP	0.117	0.052	0.121								
BLK	*0.159*	*0.096*	*0.105*	0.105							
ASI	*0.105*	0.066	0.069	0.041	0.098						
PM25	*0.118*	*0.076*	0.103	0.070	*0.081*	0.068					
PREC	0.131	0.056	0.089	0.038	0.095	0.061	0.065				
HUM	0.155	0.060	0.066	0.066	0.135	0.067	0.069	0.056			
TEMP	0.152	0.072	0.078	0.079	0.121	0.077	0.073	0.073	*0.043*		
SMK	0.118	0.059	0.092	0.035	0.092	0.051	0.064	0.037	0.052	0.065	
CAR	0.115	0.051	0.060	0.040	0.091	0.041	0.051	0.023	0.029	0.045	0.033

Note: italic: enhance, bivariate; others: enhance, nonlinear.
